# Baseline Kidney Function as Predictor of Mortality and Kidney Disease Progression in HIV-Positive Patients

**DOI:** 10.1053/j.ajkd.2012.03.006

**Published:** 2012-10

**Authors:** Fowzia Ibrahim, Lisa Hamzah, Rachael Jones, Dorothea Nitsch, Caroline Sabin, Frank A. Post, UK Collaborative HIV Cohort (CHIC)/CKD Study Group

**Affiliations:** 1 King's College London, United Kingdom; 2 Chelsea and Westminster NHS Trust, London, United Kingdom; 3 London School of Hygiene and Tropical Medicine, London, United Kingdom; 4 UCL Centre for Nephrology, Royal Free Hospital, London, United Kingdom; 5 University College London Medical School, London, United Kingdom

**Keywords:** Estimated glomerular filtration rate (eGFR), Chronic Kidney Disease Epidemiology Collaboration (CKD-EPI), human immunodeficiency virus (HIV), chronic kidney disease, mortality, competing risk

## Abstract

**Background:**

Chronic kidney disease (CKD) is associated with increased all-cause mortality and kidney disease progression. Decreased kidney function at baseline may identify human immunodeficiency virus (HIV)-positive patients at increased risk of death and kidney disease progression.

**Study Design:**

Observational cohort study.

**Setting & Participants:**

7 large HIV cohorts in the United Kingdom with kidney function data available for 20,132 patients.

**Predictor:**

Baseline estimated glomerular filtration rate (eGFR).

**Outcomes:**

Death and progression to stages 4-5 CKD (eGFR <30 mL/min/1.73 m^2^ for >3 months) in Cox proportional hazards and competing-risk regression models.

**Results:**

Median age at baseline was 34 (25th-75th percentile, 30-40) years, median CD4 cell count was 350 (25th-75th percentile, 208-520) cells/μL, and median eGFR was 100 (25th-75th percentile, 87-112) mL/min/1.73 m^2^. Patients were followed up for a median of 5.3 (25th-75th percentile, 2.0-8.9) years, during which 1,820 died and 56 progressed to stages 4-5 CKD. A U-shaped relationship between baseline eGFR and mortality was observed. After adjustment for potential confounders, eGFRs <45 and >105 mL/min/1.73 m^2^ remained associated significantly with increased risk of death. Baseline eGFR <90 mL/min/1.73 m^2^ was associated with increased risk of kidney disease progression, with the highest incidence rates of stages 4-5 CKD (>3 events/100 person-years) observed in black patients with eGFR of 30-59 mL/min/1.73 m^2^ and those of white/other ethnicity with eGFR of 30-44 mL/min/1.73 m^2^.

**Limitations:**

The relatively small numbers of patients with decreased eGFR at baseline and low rates of progression to stages 4-5 CKD and lack of data for diabetes, hypertension, and proteinuria.

**Conclusions:**

Although stages 4-5 CKD were uncommon in this cohort, baseline eGFR allowed the identification of patients at increased risk of death and at greatest risk of kidney disease progression.

Combination antiretroviral therapy (cART) has revolutionized the management of human immunodeficiency virus (HIV) infection, with dramatic decreases in the incidence of AIDS and death.^1,2^ In the developed world, the majority of deaths now are the result of nonopportunistic infections, atherosclerotic cardiovascular disease, liver and kidney disease, and non-AIDS malignancies.^3,4^ The prevalence of subclinical atherosclerosis and kidney disease is increased in HIV-positive patients.^5-10^ Although HIV-associated nephropathy is associated with high rates of kidney disease progression,^11-13^ HIV-induced immune dysregulation, inflammation, thrombotic activity, and cART toxicity may all contribute to accelerated atherosclerosis and kidney disease progression in this population.^14-18^

In the general population, proteinuria and decreased estimated glomerular filtration rate (eGFR) are risk factors for death and cardiovascular disease.^19-22^ In patients with stages 2-4 chronic kidney disease (CKD), the risk of death is much greater than the risk of developing end-stage renal disease.^23^ In HIV-positive patients, CKD similarly is associated with cardiovascular disease^24-26^ and increased risk of death,^27-31^ although the rate of progression to end-stage renal disease may be considerably higher in those of black ethnicity.^32,33^ The competing risks of mortality and kidney disease progression may differ in general and HIV-positive populations and, in the latter, by ethnicity. The aims of the present study were to examine the effect of baseline eGFR on all-cause mortality in a large HIV cohort in the United Kingdom and assess the risk of progression to stages 4-5 CKD while accounting for the competing risk of all-cause mortality.

## Methods

### Study Population and Measurements

Data were obtained from the UK Collaborative HIV Cohort (CHIC) Study.^34^ UK CHIC is an observational cohort study of HIV-positive individuals 16 years and older who have attended some of the largest HIV clinics in the United Kingdom at least once since January 1996. It is approved by the National Health Service Multi-Centre Research Ethics Committee. The present analyses include data up to December 2008 and were restricted to 7 centers that routinely contributed serum creatinine data. Information from the Office of National Statistics death register was used to ensure optimal ascertainment of deaths for patients who became lost to follow-up. Until recently, there was little recognition of the contribution of HIV or cART to non-AIDS outcomes; data for hypertension and diabetes therefore were not recorded. Similarly, data for proteinuria were not collected routinely and thus were not included in the present analyses to avoid the introduction of bias.

All available serum creatinine values were converted to eGFR using the CKD-EPI (CKD Epidemiology Collaboration) equation^35,36^: 141 × min(SCr/κ, 1)^α^ × max(SCr/κ, 1)^−1.209^ × 0.993^Age^ × 1.018 [if female] × 1.159 [if black], where SCr is serum creatinine (in milligrams per deciliter; Jaffé creatinine values were converted to isotope-dilution mass spectrometry–traceable values by multiplying by 0.95 as per Levey et al^37^); κ is 0.7 if female or 0.9 if male; α is −0.329 if female or −0.411 if male; min is the minimum of SCr/κ or 1; and max is the maximum of SCr/κ or 1.

Mortality and progression to stages 4-5 CKD (eGFR <30 mL/min/1.73 m^2^ for >3 months) were analyzed in patients stratified by baseline kidney function. Because acute renal failure is particularly common within 3 months of HIV diagnosis^38^ and the greatest changes in kidney function are observed soon after starting cART,^39^ baseline kidney function was defined as the first available eGFR that was determined more than 3 months after the time of cohort entry.

### Statistical Analysis

Data were analyzed using STATA (version 11; Stata Corp, www.stata.com). Person-years of follow-up were calculated from the date of baseline eGFR to the date of death or censoring (last clinic visit or December 31, 2008, whichever came first). Baseline parameters were compared using χ^2^, Fisher exact, or Kruskal-Wallis tests, as appropriate. Because of a significant interaction between ethnicity and eGFR (*P* < 0.001), analyses also were stratified by ethnicity (black vs white/other).

Cox proportional hazards regression models were used to examine associations between baseline eGFR and all-cause mortality, with graphical checks and Schoenfeld residual testing for the final Cox model to confirm proportionality. Baseline eGFR was stratified into 6 categories (≥105, 90-104, 60-89, 45-59, 30-44 and <30 mL/min/1.73 m^2^)^40^ and modeled as continuous piecewise linear splines with knots at 45, 60, 75, 90, and 105 mL/min/1.73 m^2^.^21^ Multivariable models were adjusted for both fixed covariates, assessed at the time of baseline eGFR (age at cohort entry, sex, HIV exposure group, and year of cohort entry) and time-updated covariates (AIDS, CD4 cell count, HIV RNA [<500 vs ≥500 copies/mL], hepatitis B and C status, and cART use [no/yes]). We took an intention-to-continue cART approach and ignored subsequent treatment interruptions. Complete or near-complete data were available for all covariates except HIV exposure group and hepatitis B and hepatitis C status. Our analyses used a missing-indicator approach to deal with missing data; thus, all patients were included in the analyses.

Competing-risk regression models^41,42^ were used to investigate associations between baseline kidney function and progression to stages 4-5 CKD because death and kidney disease progression are competing outcomes in the general CKD population.^43^ Competing-risk models provide estimations of subhazard ratios, which can be interpreted similarly to hazard ratios generated by standard Cox regression analyses. Subhazard ratios were adjusted for age at entry, sex, HIV exposure group, year of cohort entry, AIDS, CD4 cell count, HIV RNA (<500 vs ≥500 copies/mL), hepatitis B and C status, and cART use (no/yes), all as fixed covariates assessed at the time of baseline eGFR. To preserve power with enough events in each category, eGFR was stratified into 4 categories (≥90, 60-89, 45-59 and 30-44 mL/min/1.73 m^2^). Robust standard errors were used to account for the cluster effect, and all statistical tests were 2 sided.

## Results

### Baseline Characteristics

**Figure 1:**
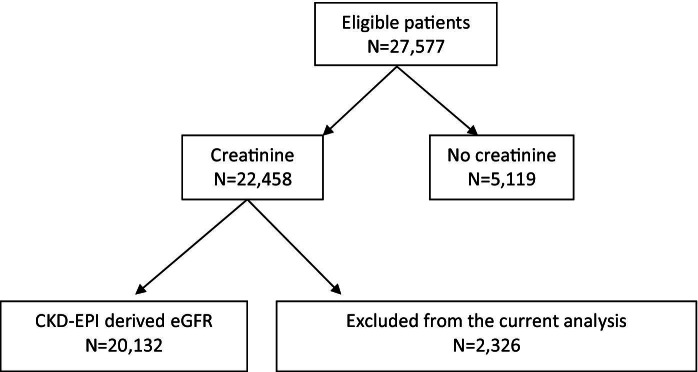
Disposition of patients for the all-cause mortality and chronic kidney disease progression analyses. Abbreviations: CKD-EPI, Chronic Kidney Disease Epidemiology Collaboration; eGFR, estimated glomerular filtration rate.

Of 27,577 patients who received care during the study period, 5,119 (19%) had no kidney function data and 2,326 (8%) died or were lost to follow-up within 3 months of cohort entry; the remaining 20,132 (73%) were included in analyses (Fig 1). Patients without kidney function data had similar CD4 cell counts at baseline but were more likely to be male, have MSM or IVDU (men who have sex with men and intravenous drug use) as risk factors for HIV acquisition, and have a lower prevalence of viral hepatitis (B and C) coinfection compared with those included in the analyses (data not shown). Baseline eGFR was assessed at a median of 4 (25th-75th percentile, 3-13) months from cohort entry and 5 (25th-75th percentile, 3-13) months from HIV diagnosis. At baseline, median age was 34 (25th-75th percentile, 30-40) years, median CD4 cell count was 350 (25th-75th percentile, 208-520) cells/μL, median eGFR was 100 (25th-75th percentile, 87-112) mL/min/1.73 m^2^, and 80% of patients had commenced cART. Table 1 lists patient characteristics stratified by baseline eGFR. Younger, black, and female patients were over-represented in those with eGFR <30 mL/min/1.73 m^2^. When stratified by ethnicity, there were more women (57%) in the black patients; 90% of white/other patients were male, the majority of whom had acquired HIV infection through sex between men. Black patients were younger (median age, 33 vs 35 years), less likely to be coinfected with hepatitis C (2.1% vs 9.6%), and had lower median CD4 cell counts (280 vs 370 cells/μL). Although black patients had a higher median eGFR compared with those of white/other ethnicity (109 vs 98 mL/min/1.73 m^2^), a greater proportion of black patients (1.1% vs 0.2%) had eGFR <30 mL/min/1.73 m^2^ at baseline.

### Baseline eGFR and All-Cause Mortality

**Figure 2:**
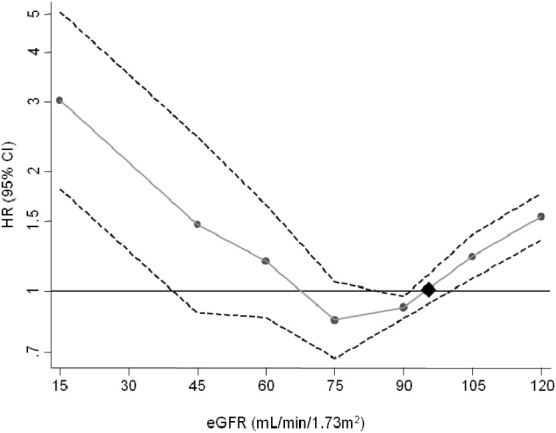
Hazard ratios (HRs) and 95% confidence intervals (CIs) for all-cause mortality and estimated glomerular filtration rate (eGFR) categories according to spline. Data were adjusted for age, sex, ethnicity, risk group, and years since entry into the cohort as fixed covariates and CD4 cell count, HIV (human immunodeficiency virus) RNA level, combination antiretroviral therapy use, AIDS, and hepatitis B surface antigen and hepatitis C antibody status as time-updated covariates. The diamond symbol represents the reference point of eGFR of 95 mL/min/1.73 m^2^ (knots at eGFRs of 45, 60, 75, 90, and 105 mL/min/1.73 m^2^).

Patients were followed up for a median of 5.3 (25th-75th percentile, 2.0-8.9) years, during which 1,820 patients (295 black and 1,525 white/other) died. The crude mortality rate in our cohort was lower for black patients than for white/other patients (1.28 [95% confidence interval (CI), 1.14-1.44] vs 1.71 [95% CI, 1.63-1.80] per 100 person-years). Table 2 lists hazard ratios for death for patients stratified by baseline eGFR. In unadjusted analyses using eGFR of 90-104 mL/min/1.73 m^2^ as the reference category, eGFR <60 or ≥105 mL/min/1.73 m^2^ at baseline was associated with increased mortality. Adjustment for demographic and HIV-associated parameters attenuated the associations between decreased eGFR and all-cause mortality, with only the eGFR categories ≥105 and <45 mL/min/1.73 m^2^ associated significantly with death (Table 2). The association between eGFR and mortality was U-shaped, with both lower and upper ends of eGFR associated with increased mortality (Fig 2). In view of a statistically significant (*P* < 0.001) interaction between eGFR and ethnicity, we repeated the analysis stratified by ethnicity (Table S1, available as online supplementary material). The U-shaped relationship was present in patients of both black and white/other ethnicity, with a more pronounced increase in mortality risk with decreasing eGFR in black patients.

### Baseline eGFR and Progression to Stages 4-5 CKD

By the end of the study period, 118 (0.6%) patients had stages 4-5 CKD. In 62 of these patients (53%), stages 4-5 CKD were already established at baseline. Patients with stages 4-5 CKD had a median age of 38 (25th-75th percentile, 33-45) years, were predominantly male (69%) and of black ethnicity (54%), had low rates of hepatitis B or C coinfection (9% and 4%, respectively), and had more advanced immunodeficiency at baseline (median CD4 cell count, 213 [25th-75th percentile, 93-365] cells/μL compared with 348 [25th-75th percentile, 203-520] cells/μL in those not categorized as CKD stages 4-5).

To examine the relationship between baseline eGFR and kidney disease progression, we used Cox models that allowed adjustments for the competing risk of death. Standard time-to-event analyses assume that censoring over time (drop out of the study over time) is independent of both exposure and outcome. For kidney disease progression, these models would assume that deaths are independent of kidney disease and, because our previous analyses have shown that the exposure of interest (eGFR) and ethnicity are predictors of death, thus may be biased. The competing-risk model allows for death to be correlated with kidney disease progression; in other words, the fitted model allows for kidney disease progressors to have a higher mortality risk. Hence, we used competing-risk models, and results for the association of eGFR with kidney disease progression are expressed as subhazard ratios.

All 20,045 patients with eGFR ≥30 mL/min/1.73 m^2^ at baseline were included in the competing-risk regression analysis. Median number of available eGFR determinations was 18 (25th-75th percentile, 8-32) per patient and median interval between measurements was 3.5 (25th-75th percentile, 2.3-5.6) months. Progression to stages 4-5 CKD was observed in 0.1%, 0.33%, 3.5%, and 23.1% of patients with baseline eGFR ≥90, 60-89, 45-59, and 30-44 mL/min/1.73 m^2^, respectively, with a median interval of 1.39 (25th-75th percentile, 0.18-4.59) years between baseline and the first sustained eGFR <30 mL/min/1.73 m^2^. Progression to stages 4-5 CKD was more frequent in black compared with white/other patients (crude incidence rates of 0.92 [95% CI, 0.61-1.39] vs 0.33 [95% CI, 0.23-0.46] per 1,000 person-years; rate ratio, 2.8 [95% CI, 1.6-4.8]; *P* = 0.001), and increased from 0.16 (95% CI, 0.09-0.28) to 0.47 (95% CI, 0.30-0.75), 5.0 (95% CI, 2.61-9.62), and 47.4 (95% CI, 28.58-78.62) per 1,000 person-years in patients with baseline eGFR ≥90, 60-89, 45-59, and 30-44 mL/min/1.73 m^2^, respectively. Interestingly, the incidence of stages 4-5 CKD in patients with eGFR of 60-89 mL/min/1.73 m^2^ at baseline was nearly 10-fold higher in black compared with white/other patients (2.13 vs 0.24/1,000 person-years).

In both unadjusted and adjusted analyses, baseline eGFR was associated strongly with progression to stages 4-5 CKD (Table 3). The association of eGFR with kidney disease progression differed by ethnicity (Table 4). In black patients, the subhazard ratio for progression to stages 4-5 CKD increased with decreasing baseline eGFR <90 mL/min/1.73 m^2^, whereas in white/other patients, the association with progression was detectable only with decreasing baseline eGFR <60 mL/min/1.73 m^2^. These hazards were affected minimally by adjustment for demographic and HIV-associated parameters. However, the absolute risk of stages 4-5 CKD in black patients with eGFR of 60-89 mL/min/1.73 m^2^ and white/other patients with eGFR of 45-59 mL/min/1.73 m^2^ was low (2.1 and 2.6 events/1,000 person-years). In contrast, high rates of progression to stages 4-5 CKD were observed in black patients with eGFR of 30-59 mL/min/1.73 m^2^ and white/other patients with eGFR of 30-44 mL/min/1.73 m^2^ (31.1 and 37.0 events/1,000 person-years, respectively).

## Discussion

In this large HIV cohort, we observed an independent U-shaped relationship between baseline eGFR and mortality, with the highest risk in those with stages 4-5 CKD at baseline. In addition, in analyses that adjusted for the competing mortality risk, baseline eGFR was an important predictor of kidney disease progression. Decreased eGFR at baseline was of much greater prognostic significance in black patients in terms of both death and kidney disease progression. With incidence rates of stages 4-5 CKD >3%, black patients with eGFR of 30-59 mL/min/1.73 m^2^ and white/other patients with eGFR of 30-44 mL/min/1.73 m^2^ at baseline should be investigated, monitored carefully, and considered for targeted interventions to slow the decrease in kidney function.

Several studies have examined the effect on mortality when patients have decreased kidney function prior to the initiation of cART. In HIV-positive women, prevalent CKD, defined as eGFR <60 mL/min/1.73 m^2^ at 2 consecutive visits, was an independent risk factor for death.^27^ In a large African cohort from Zambia, eGFR <90 mL/min/1.73 m^2^ prior to initiation of cART was associated with increased risk of death, with the highest risk in those with eGFR <30 mL/min/1.73 m^2^,^28^ whereas in the FRAM (Fat Redistribution and Metabolic Change in HIV) Study, a creatinine-based eGFR <60 mL/min/1.73 m^2^ was associated with death in only univariate analysis.^29^ Although these studies have clearly demonstrated a relationship between decreased eGFR and mortality, the observed association was attenuated in analyses that were able to adjust for diabetes or hypertension^27^ or albuminuria, smoking, dyslipidemia, and body morphology.^29^

In the Zambian study, more than half of all deaths were observed in the first 3 months from cohort entry.^28^ Decreased eGFR prior to initiation of cART may reflect both acute kidney injury and CKD.^11,38^ Changes in eGFR are observed predominantly during early exposure to cART, with stabilization of kidney function after ∼4 weeks.^39^ Consequently, we excluded kidney function data and deaths that occurred within the first 3 months of HIV diagnosis, and our estimates of baseline eGFR are likely to reflect kidney function in the steady state. Consistent with results from the FRAM Study,^29^ we observed no association between eGFR of 30-59 mL/min/1.73 m^2^ and mortality in a predominantly white cohort of more than 15,000 patients.

We observed a U-shaped relationship between eGFR and death. Consistent with observations in the general population,^21^ mortality was lowest in patients with eGFR of 90-105 mL/min/1.73 m^2^, whereas those with eGFR ≥105 mL/min/1.73 m^2^ were at significantly increased risk of death. However, eGFR prediction equations are inaccurate at high eGFRs, and high eGFR may reflect glomerular hyperfiltration^44^ or ill health in patients with decreased muscle mass.^21,45^ This is supported by the linear relationship between an alternative marker of kidney function, cystatin C (which does not depend on muscle mass), and mortality.^21^

The risk of kidney disease progression in HIV-positive black patients is increased,^32,46-49^ and recent genetic studies implicate the *APOL1* G1 and G2 alleles to account in part for this excess of risk.^50-52^ However, the absolute risk of stages 4-5 CKD in this population was low, with ∼1% of participants in our study reaching this end point. Consistent with our earlier studies of HIV-associated kidney disease,^11,33^ more than half of those with stages 4-5 CKD already had eGFR <30 mL/min/1.73 m^2^ at baseline, and many of these patients are likely to have had irreversible kidney damage at the time of HIV diagnosis.^11^

Data for the role of cART in kidney disease progression are conflicting; several antiretrovirals, including tenofovir, indinavir, and atazanavir, have been associated with kidney disease progression.^26,53^ However, immunodeficiency, HIV viremia, and nonuse of cART are common factors in patients with stages 4-5 CKD,^11,33,54,55^ and the use of cART and suppression of HIV RNA may improve kidney function,^56^ reduce the rate of eGFR decrease,^57^ or reduce the risk of renal events.^58^ Our data suggest that in addition to these factors, baseline eGFR is helpful in assessing the risk of developing stages 4-5 CKD in HIV-positive patients.

The strengths of this study include the large sample size and prolonged follow-up. However, this study was conducted in a population at relatively low risk of CKD (predominately white and with a low hepatitis C prevalence). Limitations therefore include the relatively small numbers of patients with decreased eGFR at baseline and the low rates of progression to stages 4-5 CKD, limiting the power of the study and resulting in large 95% CIs around point estimates in the competing-risk analysis. We are unable to account for loss to follow-up, including those referred to clinics not participating in CHIC. Furthermore, we lacked data for cardiovascular and renal risk factors, such as diabetes, hypertension, and proteinuria; a reliable indicator of muscle mass; clinical status; socioeconomic status; and information for cause of death. Finally, we have not accounted for small differences in creatinine calibration between contributing laboratories.

In summary, our results show that decreased eGFR at baseline is an independent risk factor for all-cause mortality and progression to stages 4-5 CKD. The low rates of kidney disease progression observed in our cohort are explained at least in part by considerable competing mortality. Our results highlight the importance of early HIV diagnosis because stages 4-5 CKD were already established at baseline in most patients. Because black HIV-positive patients with eGFR of 30-59 mL/min/1.73 m^2^ and white/other HIV-positive patients with eGFR of 30-44 mL/min/1.73 m^2^ were at high risk of kidney disease progression, their eGFR should be monitored closely during clinical follow-up.

## Figures and Tables

**Table 1:** Baseline Characteristics in Patients Stratified by eGFR Category

Variable	eGFR Category (mL/min/1.73 m^2^)						*P*
	≥105 (n = 8,307; 41%)	104-90 (n = 5,951; 30%)	89-60 (n = 5,466; 27%)	59-45 (n = 256; 1.3%)	44-30 (n = 65; 0.3%)	<30 (n = 87; 0.4%)	
Age at cohort entry (y)	32 [28, 36]	35 [30, 41]	38 [33, 46]	44 [36, 54]	41 [35, 50]	38 [33, 45]	<0.001
Sex							<0.001
Female	2,156 (26.0)	1,036 (17.0)	1,015 (18.6)	54 (21.1)	19 (29.2)	37 (42.5)	
Male	6,151 (74.0)	4,915 (83.0)	4,451 (81.4)	202 (78.9)	46 (70.8)	50 (57.5)	
Ethnicity							<0.001
Black	2,838 (34.2)	1,158 (19.5)	877 (16.0)	50 (19.5)	25 (38.5)	54 (62.1)	
White/other	5,469 (65.8)	4,793 (80.5)	4,589 (84.0)	206 (80.5)	40 (61.5)	33 (37.9)	
Risk group							<0.001
IVDU/other	800 (9.6)	412 (6.9)	325 (6.0)	23 (9.0)	7 (10.8)	16 (18.4)	
MSM	4,402 (53.0)	3,871 (65.0)	3,503 (64.1)	131 (51.2)	23 (35.4)	15 (17.2)	
Heterosexual	2,786 (33.5)	1,433 (24.1)	1,334 (24.4)	73 (28.5)	30 (46.2)	53 (60.9)	
HBsAg+	449 (5.4)	327 (5.5)	275 (5.0)	9 (3.5)	1 (1.5)	3 (3.4)	0.4
HCV Ab+	634 (7.6)	486 (8.2)	407 (7.5)	21 (8.2)	4 (6.2)	1 (1.1)	0.1
AIDS^a^	2,222 (26.7)	1,746 (29.3)	1,720 (31.5)	107 (41.8)	26 (40.0)	34 (39.1)	<0.001
CD4 cell count (cells/μL)	350 [206, 518]	360 [217, 532]	334 [196, 511]	220 [130, 383]	160 [84, 289]	202 [96, 330]	<0.001
HIV RNA (copies/mL)	6,622 [184, 50,500]	8,380 [326, 57,000]	5,147 [200, 46,000]	822 [50, 28,330]	500 [50, 46,000]	577 [50, 59,065]	<0.001
Initiated cART	6,545 (78.8)	4,714 (79.2)	4,487 (82.1)	220 (85.9)	56 (86.2)	77 (88.5)	<0.001

*Note:* Continuous variables given as median [25th, 75th percentile]; categorical variables given as number (column percentage). *P* values are unadjusted, using χ^2^ tests for difference in proportions and Kruskal-Wallis tests for difference in median values. Abbreviations and definitions: cART, combination antiretroviral therapy; eGFR, estimated glomerular filtration rate; HBsAg+, hepatitis B surface antigen positive; HCV Ab+, hepatitis C antibody positive; HIV, human immunodeficiency virus; IVDU, intravenous drug use; MSM, men who have sex with men.

a Per Centers for Disease Control and Prevention classification system for HIV-infected adults and adolescents.

**Table 2:** Mortality Rates by eGFR Category and Association of eGFR Category With All-Cause Mortality

Baseline eGFR	Events^a^	Crude		Adjusted^b^	
		HR (95% CI)	*P*	HR (95% CI)	*P*
≥105 mL/min/1.73 m^2^	703/41,385	1.15 (1.03-1.30)	0.01	1.31 (1.17-1.47)	<0.001
90-104 mL/min/1.73 m^2^	502/34,862	1.00 (reference)		1.00 (reference)	
60-89 mL/min/1.73 m^2^	520/33,838	1.08 (0.95-1.22)	0.2	0.92 (0.81-1.04)	0.2
45-59 mL/min/1.73 m^2^	56/1,465	2.65 (2.01-3.50)	<0.001	1.34 (0.95-1.89)	0.09
30-44 mL/min/1.73 m^2^	15/257	3.88 (2.32-6.48)	<0.001	1.70 (1.06-2.72)	0.03
<30 mL/min/1.73 m^2^	24/260	5.75 (3.81-8.66)	<0.001	3.08 (1.95-4.88)	<0.001

*Note:* A statistically significant interaction (*P* < 0.001) between eGFR and ethnicity was present. Abbreviations: CI, confidence interval; eGFR, estimated glomerular filtration rate; HR, hazard ratio.

a Reported per person-years of follow-up.

b Adjusted for age, sex, ethnicity, risk group, and years since entry into the cohort; as well as CD4 cell count, HIV RNA level, combination antiretroviral therapy use, AIDS, hepatitis B surface antigen, and hepatitis C antibody status as time-updated covariates.

**Table 3:** Association of Baseline eGFR Category With Progression to Stages 4-5 CKD

Baseline eGFR	No. of eGFRs^a^	Events^b^	Crude		Adjusted^c^	
			SHR (95% CI)	*P*	SHR (95% CI)	*P*
≥90 mL/min/1.73 m^2^	17 [7, 31]	14/84,853	1.00 (reference)		1.00 (reference)	
60-89 mL/min/1.73 m^2^	20 [8, 34]	18/37,763	2.95 (1.47-5.92)	0.002	3.51 (1.57-7.86)	0.002
45-59 mL/min/1.73 m^2^	23 [10, 41]	9/1,797	29.9 (12.9-69.2)	<0.001	39.0 (15.0-101.3)	<0.001
30-44 mL/min/1.73 m^2^	18 [12, 41]	15/317	280.5 (132.8-592.5)	<0.001	361.3 (151.1-864.3)	<0.001

*Note:* Analysis adjusted for competing end point of all-cause mortality. Abbreviations: CI, confidence interval; CKD, chronic kidney disease; eGFR, estimated glomerular filtration rate; SHR, subdistribution hazard ratio.

a Total number of times eGFR was determined during follow-up (excludes baseline); values given as median [25th, 75th percentile].

b Reported per person-years of follow-up.

c Adjusted for age, sex, risk group, ethnicity, years since entry into the cohort, CD4 cell count, HIV RNA level, combination antiretroviral therapy use, and AIDS; as well as hepatitis B surface antigen and hepatitis C antibody status at baseline (measurements taken at same time as the baseline eGFR).

**Table 4:** Association of Baseline eGFR Category With Progression to Stages 4-5 CKD, Stratified by Ethnicity

eGFR^a^	Black					White/Other				
	Events^b^	Crude		Adjusted^c^		Events^b^	Crude		Adjusted^c^	
		SHR (95% CI)	*P*	SHR (95% CI)	*P*		SHR (95% CI)	*P*	SHR (95% CI)	*P*
≥90	2/19,822	1.00 (reference)		1.00 (reference)		12/65,031	1.00 (reference)		1.00 (reference)	
60-89	10/4,699	21.8 (4.7-100.0)	<0.001	26.4 (4.7-148.9)	<0.001	8/33,064	1.3 (0.5-3.2)	0.5	1.2 (0.5-3.1)	0.7
45-59	5/280	177.4 (33.0-954.0)	<0.001	196.7 (31.5-1,229.4)	<0.001	4/1,517	13.9 (4.6-42.1)	<0.001	16.2 (4.2-62.1)	<0.001
30-44	6/74	730.2 (155.8-3,423.5)	<0.001	1,163.4 (217.0-6,237.6)	<0.001	9/243	194.5 (78.0-485.1)	<0.001	217.7 (63.7-743.5)	<0.001

*Note:* Analysis adjusted for competing end point of all-cause mortality. Abbreviations: CI, confidence interval; CKD, chronic kidney disease; eGFR, estimated glomerular filtration rate; SHR, subdistribution hazard ratio.

a eGFR at baseline (given in mL/min/1.73 m^2^).

b Reported per person-years of follow-up.

c Adjusted for age, sex, risk group, years since entry into the cohort, CD4 cell count, HIV (human immunodeficiency virus) RNA level, combination antiretroviral therapy use, and AIDS, as well as hepatitis B surface antigen and hepatitis C antibody status at baseline.

## Supplementary Material

Supplementary Table S1 (PDF) Mortality rates by baseline eGFR and ethnicity, and associations of eGFR with all-cause mortality, stratified by ethnicity.

## References

[1] Palella F.J., Delaney K.M., Moorman A.C. (1998). Declining morbidity and mortality among patients with advanced human immunodeficiency virus infection: HIV Outpatient Study Investigators. N Engl J Med.

[2] Mocroft A., Ledergerber B., Katlama C. (2003). Decline in the AIDS and death rates in the EuroSIDA study: an observational study. Lancet.

[3] Marin B., Thiebaut R., Bucher H.C. (2009). Non-AIDS-defining deaths and immunodeficiency in the era of combination antiretroviral therapy. AIDS.

[4] Antiretroviral Therapy Cohort Collaboration (2010). Causes of death in HIV-1-infected patients treated with antiretroviral therapy, 1996-2006: collaborative analysis of 13 HIV cohort studies. Clin Infect Dis.

[5] Grunfeld C., Delaney J.A., Wanke C. (2009). Preclinical atherosclerosis due to HIV infection: carotid intima-medial thickness measurements from the FRAM Study. AIDS.

[6] Hsue P.Y., Hunt P.W., Schnell A. (2009). Role of viral replication, antiretroviral therapy, and immunodeficiency in HIV-associated atherosclerosis. AIDS.

[7] Kingsley L.A., Cuervo-Rojas J., Munoz A. (2008). Subclinical coronary atherosclerosis, HIV infection and antiretroviral therapy: Multicenter AIDS Cohort Study. AIDS.

[8] Fitch K.V., Lo J., Abbara S. (2010). Increased coronary artery calcium score and noncalcified plaque among HIV-infected men: relationship to metabolic syndrome and cardiac risk parameters. J Acquir Immune Defic Syndr.

[9] Post F.A., Holt S.G. (2009). Recent developments in HIV and the kidney. Curr Opin Infect Dis.

[10] Szczech L.A., Grunfeld C., Scherzer R. (2007). Microalbuminuria in HIV infection. AIDS.

[11] Post F.A., Campbell L.J., Hamzah L. (2008). Predictors of renal outcome in HIV-associated nephropathy. Clin Infect Dis.

[12] Szczech L.A., Gupta S.K., Habash R. (2004). The clinical epidemiology and course of the spectrum of renal diseases associated with HIV infection. Kidney Int.

[13] Atta M.G., Lucas G.M., Fine D.M. (2008). HIV-associated nephropathy: epidemiology, pathogenesis, diagnosis and management. Expert Rev Anti Infect Ther.

[14] Neuhaus J., Jacobs D.R., Baker J.V. (2010). Markers of inflammation, coagulation, and renal function are elevated in adults with HIV infection. J Infect Dis.

[15] Deeks S.G., Phillips A.N. (2009). HIV infection, antiretroviral treatment, ageing, and non-AIDS related morbidity. BMJ.

[16] Kuller L.H., Tracy R., Belloso W. (2008). Inflammatory and coagulation biomarkers and mortality in patients with HIV infection. PLoS Med.

[17] Baker J.V., Neuhaus J., Duprez D. (2011). Changes in inflammatory and coagulation biomarkers: a randomized comparison of immediate versus deferred antiretroviral therapy in patients with HIV infection. J Acquir Immune Defic Syndr.

[18] Tien P.C., Choi A.I., Zolopa A.R. (2010). Inflammation and mortality in HIV-infected adults: analysis of the FRAM Study cohort. J Acquir Immune Defic Syndr.

[19] Muntner P., He J., Hamm L., Loria C., Whelton P.K. (2002). Renal insufficiency and subsequent death resulting from cardiovascular disease in the United States. J Am Soc Nephrol.

[20] Tonelli M., Wiebe N., Culleton B. (2006). Chronic kidney disease and mortality risk: a systematic review. J Am Soc Nephrol.

[21] Matsushita K., van der Velde M., Astor B.C. (2010). Association of estimated glomerular filtration rate and albuminuria with all-cause and cardiovascular mortality in general population cohorts: a collaborative meta-analysis. Lancet.

[22] Go A.S., Chertow G.M., Fan D., McCulloch C.E., Hsu C.Y. (2004). Chronic kidney disease and the risks of death, cardiovascular events, and hospitalization. N Engl J Med.

[23] Keith D.S., Nichols G.A., Gullion C.M., Brown J.B., Smith D.H. (2004). Longitudinal follow-up and outcomes among a population with chronic kidney disease in a large managed care organization. Arch Intern Med.

[24] Choi A.I., Li Y., Deeks S.G., Grunfeld C., Volberding P.A., Shlipak M.G. (2010). Association between kidney function and albuminuria with cardiovascular events in HIV-infected persons. Circulation.

[25] George E., Lucas G.M., Nadkarni G.N., Fine D.M., Moore R., Atta M.G. (2010). Kidney function and the risk of cardiovascular events in HIV-1-infected patients. AIDS.

[26] Campbell L.J., Ibrahim F., Fisher M., Holt S.G., Hendry B.M., Post F.A. (2009). Spectrum of chronic kidney disease in HIV-infected patients. HIV Med.

[27] Estrella M.M., Parekh R.S., Abraham A. (2010). The impact of kidney function at highly active antiretroviral therapy initiation on mortality in HIV-infected women. J Acquir Immune Defic Syndr.

[28] Mulenga L.B., Kruse G., Lakhi S. (2008). Baseline renal insufficiency and risk of death among HIV-infected adults on antiretroviral therapy in Lusaka, Zambia. AIDS.

[29] Choi A., Scherzer R., Bacchetti P. (2010). Cystatin C, albuminuria, and 5-year all-cause mortality in HIV-infected persons. Am J Kidney Dis.

[30] Atta M.G., Fine D.M., Kirk G.D., Mehta S.H., Moore R.D., Lucas G.M. (2007). Survival during renal replacement therapy among African Americans infected with HIV type 1 in urban Baltimore, Maryland. Clin Infect Dis.

[31] Gardner L.I., Holmberg S.D., Williamson J.M. (2003). Development of proteinuria or elevated serum creatinine and mortality in HIV-infected women. J Acquir Immune Defic Syndr.

[32] Choi A.I., Rodriguez R.A., Bacchetti P., Bertenthal D., Volberding P.A., O'Hare A.M. (2007). Racial differences in end-stage renal disease rates in HIV infection versus diabetes. J Am Soc Nephrol.

[33] Bansi L., Hughes A., Bhagani S. (2009). Clinical epidemiology of HIV-associated end-stage renal failure in the UK. AIDS.

[34] UK Collaborative HIV Cohort Steering Committee (2004). The creation of a large UK-based multicentre cohort of HIV-infected individuals: the UK Collaborative HIV Cohort (UK CHIC) Study. HIV Med.

[35] Levey A.S., Stevens L.A., Schmid C.H. (2009). A new equation to estimate glomerular filtration rate. Ann Intern Med.

[36] Ibrahim F., Hamzah L., Jones R., Nitsch D., Sabin C., Post F.A. (2011). Comparison of CKD-EPI and MDRD to estimate baseline renal function in HIV-positive patients [published online ahead of print November 25, 2011]. Nephrol Dial Transplant.

[37] Levey A.S., Coresh J., Greene T. (2007). Expressing the Modification of Diet in Renal Disease Study equation for estimating glomerular filtration rate with standardized serum creatinine values. Clin Chem.

[38] Roe J., Campbell L.J., Ibrahim F., Hendry B.M., Post F.A. (2008). HIV care and the incidence of acute renal failure. Clin Infect Dis.

[39] Reid A., Stohr W., Walker A.S. (2008). Severe renal dysfunction and risk factors associated with renal impairment in HIV-infected adults in Africa initiating antiretroviral therapy. Clin Infect Dis.

[40] National Kidney Foundation (2002). KDOQI Clinical Practice Guidelines for Chronic Kidney Disease: evaluation, classification, and stratification. http://www.kidney.org/professionals/KDOQI/guidelines_ckd/toc.htm.

[41] Lunn M., McNeil D. (1995). Applying Cox regression to competing risks. Biometrics.

[42] Shiels M.S., Cole S.R., Chmiel J.S. (2010). A comparison of ad hoc methods to account for non-cancer AIDS and deaths as competing risks when estimating the effect of HAART on incident cancer AIDS among HIV-infected men. J Clin Epidemiol.

[43] Agarwal R., Bunaye Z., Bekele D.M., Light R.P. (2008). Competing risk factor analysis of end-stage renal disease and mortality in chronic kidney disease. Am J Nephrol.

[44] Succi R, Gouvea A, Machado DM, et al. Can glomerular hyperfiltration measurement predict later development of HIVassociated nephropathy? Poster presented at: 28th Annual Meeting of the European Society for Paediatric Infectious Diseases; May 4-8, 2010; Nice, France.

[45] Cox H.J., Bhandari S., Rigby A.S., Kilpatrick E.S. (2008). Mortality at low and high estimated glomerular filtration rate values: a ‘U’ shaped curve. Nephron Clin Pract.

[46] Lucas G.M., Lau B., Atta M.G., Fine D.M., Keruly J., Moore R.D. (2008). Chronic kidney disease incidence, and progression to end-stage renal disease, in HIV-infected individuals: a tale of two races. J Infect Dis.

[47] Lucas G.M., Mehta S.H., Atta M.G. (2007). End-stage renal disease and chronic kidney disease in a cohort of African-American HIV-infected and at-risk HIV-seronegative participants followed between 1988 and 2004. AIDS.

[48] Lucas G.M., Clarke W., Kagaayi J. (2010). Decreased kidney function in a community-based cohort of HIV-infected and HIV-negative individuals in Rakai, Uganda. J Acquir Immune Defic Syndr.

[49] Choi A.I., Rodriguez R.A., Bacchetti P., Bertenthal D., Volberding P.A., O'Hare A.M. (2007). The impact of HIV on chronic kidney disease outcomes. Kidney Int.

[50] Papeta N., Kiryluk K., Patel A. (2011). APOL1 variants increase risk for FSGS and HIVAN but not IgA nephropathy. J Am Soc Nephrol.

[51] Kopp J.B., Nelson G.W., Sampath K. (2011). APOL1 genetic variants in focal segmental glomerulosclerosis and HIV-associated nephropathy. J Am Soc Nephrol.

[52] Fine D.M., Wasser W.G., Estrella M.M. (2012). APOL1 risk variants predict histopathology and progression to ESRD in HIV-related kidney disease. J Am Soc Nephrol.

[53] Mocroft A., Kirk O., Reiss P. (2010). Estimated glomerular filtration rate, chronic kidney disease and antiretroviral drug use in HIV-positive patients. AIDS.

[54] Lucas G.M., Eustace J.A., Sozio S., Mentari E.K., Appiah K.A., Moore R.D. (2004). Highly active antiretroviral therapy and the incidence of HIV-1-associated nephropathy: a 12-year cohort study. AIDS.

[55] Choi A., Rodriguez R., Bacchetti P. (2007). Low rates of antiretroviral therapy among HIV-infected patients with chronic kidney disease. Clin Infect Dis.

[56] Peters P.J., Moore D.M., Mermin J. (2008). Antiretroviral therapy improves renal function among HIV-infected Ugandans. Kidney Int.

[57] Choi A.I., Shlipak M.G., Hunt P.W., Martin J.N., Deeks S.G. (2009). HIV-infected persons continue to lose kidney function despite successful antiretroviral therapy. AIDS.

[58] El-Sadr W.M., Lundgren J.D., Neaton J.D. (2006). CD4^+^ count-guided interruption of antiretroviral treatment. N Engl J Med.

