# Supplementary material for: Baseline Kidney Function as Predictor of Mortality and Kidney Disease Progression in HIV-Positive Patients
Source: Am J Kidney Dis. 2012 Oct;60(4):539–47. doi: 10.1053/j.ajkd.2012.03.006 (PMC3657190; doi:10.1053/j.ajkd.2012.03.006)
Supplement: Supplementary Table S1 (PDF) — Mortality rates by baseline eGFR and ethnicity, and associations of eGFR with all-cause mortality, stratified by ethnicity. [file mmc1.pdf]

Table S1: Crude mortality rates by baseline eGFR and ethnicity, and crude and adjusted associations of eGFR with all-cause mortality stratified by ethnicity

	Black					White/other				
eGFR ($ml/min/1.73m^2$)	Events/PYs	Crude HR (95% CI)	p-value	Adjusted* HR (95% CI)	p-value	Events/PYs	Crude HR (95% CI)	p-value	Adjusted* HR (95% CI)	p-value
≥105	158/12692	1.44 (1.04, 1.98)	0.03	1.53 (1.08, 2.18)	0.02	545/28693	1.20 (1.06, 1.36)	0.01	1.53 (1.32, 1.77)	<0.001
90-104	48/5639	reference		reference		454/29223	reference		reference	
60-89	59/4261	1.63 (1.11, 2.38)	0.01	1.47 (0.97, 2.22)	0.07	461/29577	1.01 (0.89, 1.15)	0.87	0.88 (0.76, 1.02)	0.09
45-59	14/211	7.56 (4.23, 13.5)	<0.001	4.17 (2.42,7.18)	<0.001	42/1254	2.17 (1.56, 3.01)	<0.001	1.09 (0.73, 1.62)	0.67
30-44	3/59	5.17 (1.58, 17.0)	0.01	2.16 (0.68, 6.84)	0.19	12/198	3.83 (2.08, 7.06)	<0.001	1.84 (1.12, 3.02)	0.02
<30	13/147	9.23 (5.03, 16.9)	<0.001	4.67 (2.36, 9.24)	<0.001	11/113	5.78 (3.16, 10.6)	<0.001	3.00 (1.11, 8.10)	0.03

*Adjusted for age, gender, risk group, years since entry into the cohort, and CD4 cell count, HIV RNA level, cART use, AIDS, HBsAg and HCV Ab status as time-updated covariates

cART, combination antiretroviral therapy; HBsAg, hepatitis B surface antigen; HCV Ab, hepatitis C antibody; eGFR, estimated glomerular filtration rate; CI, confidence intervals; HR, hazard ratio; PYs, person years of follow-up
